# Primary intrathoracic liposarcoma: a clinical analysis of 31 cases

**DOI:** 10.1186/s40880-019-0358-8

**Published:** 2019-04-02

**Authors:** Zhixue Fu, Kun Yang, Xu Yang, Shulan Chen, Wenqing Wang, Dongfu Chen, Jun Zhao, Zhishan Li, Qinfu Feng, Zongmei Zhou, Luhua Wang, Shugeng Gao, Jun Liang

**Affiliations:** 1 Department of Radiation Oncology, National Cancer Center/Cancer Hospital, Chinese Academy of Medical Sciences and Peking Union Medical College, Beijing, 100021 P. R. China; 2 Department of Thoracic Surgery, National Cancer Center/Cancer Hospital, Chinese Academy of Medical Sciences and Peking Union Medical College, Beijing, 100021 P. R. China; 3 Department of Medical Records, National Cancer Center/Cancer Hospital, Chinese Academy of Medical Sciences and Peking Union Medical College, Beijing, 100021 P. R. China

Dear Editor,

Liposarcoma is a common soft tissue malignancy in adults. It most commonly occurs in the deep soft tissue of the extremities and retroperitoneum. Primary intrathoracic liposarcoma is rare, represents 2.7% of all liposarcomas [1]. According to the distinct tumor locations, it can be divided into mediastinal, pleural, and pulmonary liposarcomas. Most literature has reviewed the clinicopathological or molecular cytogenetic characteristics of mediastinal and thoracic liposarcomas [2, 3]. However, intrathoracic liposarcoma is poorly documented because of its rarity. Complete surgical resection is thought to be the best treatment for primary intrathoracic liposarcoma, but the impact of radiotherapy and systemic therapy remains unclear. Therefore, we analyzed the clinicopathological data of 31 patients with primary intrathoracic liposarcoma who were treated in the Cancer Hospital of Chinese Academy of Medical Sciences between October 1970 and July 2015 to explore their clinicopathologic features and treatment outcomes (Additional file 1: Table S1).

## Clinical characteristics

In the present study, the median age of the 31 patients with primary intrathoracic liposarcoma was 45 years (range 20–64 years), which was the same as the age of patients with primary extremity liposarcoma reported by Moreau et al. [4]. However, the mean ages of primary intrathoracic liposarcoma patients reported by the other two studies were 43 and 58 years [2, 5].

We found that the most common initial symptom of intrathoracic liposarcoma was chest tightness (13 cases, 41.9%). Additionally, 6 (19.4%) patients were asymptomatic. However, Burt et al. [3] reported that the most common symptoms of patients with mediastinal liposarcoma were chest pain, dyspnea, wheezing, cough, and weight loss. Dei et al. [6] reported that mediastinal liposarcomas were often asymptomatic until they reached an appreciable size, at which point the symptoms were caused by direct invasion or compression of the heart, great vessels, or lungs.

We also observed that the percentage of patients with tumors located in mediastinum was 77.4% (24 cases), which was much higher than that (37.5%) reported by Hahn and Fletcher [5]. Of the 24 patients with mediastinal liposarcomas, 8 (33.3%) had tumors located in the posterior mediastinum, and 16 (66.7%) in the anterior mediastinum. However, Gladish et al. [7] reported that intrathoracic soft tissue sarcomas were often found in the lungs, mediastinum, pleura, pericardium, heart, and chest wall, whereas Unal et al. [8] found that they were most frequently localized on the chest wall and lungs.

The median tumor diameter was 10.0 cm (range 1.8–32.0 cm). The World Health Organization classification of soft tissue tumors recognizes five categories of liposarcomas: well-differentiated liposarcoma/atypical lipomatous tumor, dedifferentiated liposarcoma, myxoid liposarcoma, pleomorphic liposarcoma, and mixed-type liposarcoma. The most common histological subtype was myxoid liposarcoma (13 cases, 41.9%), followed by well-differentiated liposarcoma (6 cases, 19.4%), whereas dedifferentiated liposarcoma (3 cases 9.7%) was the rarest subtype in the present study. Chen et al. [9] also reported that the most common subtypes were myxoid liposarcoma (34.8%) and well-differentiated liposarcoma (34.8%). Enzinger et al. [10] reported that 40.0%–50.0% of mediastinal liposarcoma was well-differentiated liposarcoma, and 20.0%–30.0% was myxoid liposarcoma.

## Treatment

In the present study, 28 (90.3%) patients had undergone surgery: 21 (75.0%) with complete (R0) resection and 7 (25.0%) with microscopic residual tumor (R1) or macroscopic residual tumor (R2) resection. The R0 resection rate reported by Hahn and Fletcher [3] was 54.2%. Surgical removal is considered to be the best treatment for liposarcomas, including primary mediastinal liposarcoma.

Thirteen (41.9%) patients had received radiotherapy with total dose from 20 to 66 Gy: 2 (15.4%) had neoadjuvant radiotherapy, 8 (61.5%) had adjuvant radiotherapy and 3 (23.1%) had radiotherapy alone. Additionally, 5 (38.5%) patients had two-dimensional radiotherapy, 2 (15.4%) had three-dimensional conformal radiotherapy, and 6 (46.2%) had intensity-modulated radiotherapy. Among the 8 patients who had received adjuvant radiotherapy, 3 (37.5%) also had R1 or R2 resection, and 5 (62.5%) had R0 resection.

Three patients had received chemotherapy: 1 (33.3%) had adjuvant chemotherapy and 2 (66.7%) had neoadjuvant chemoradiotherapy. The chemotherapy regimens used to treat these patients included adriamycin plus ifosfamide and epirubicin plus ifosfamide.

## Survival outcomes

**Fig. 1:**
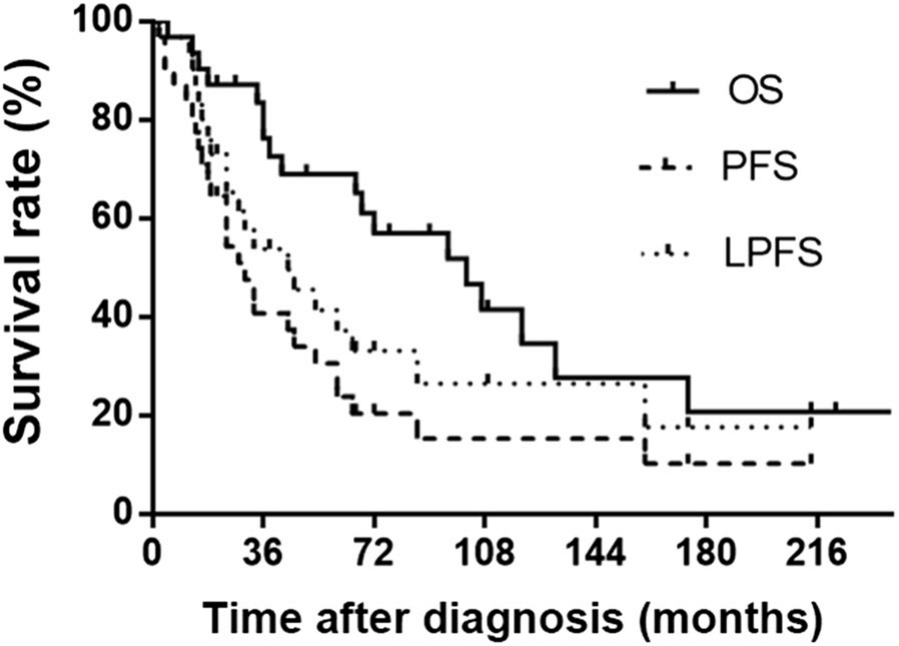
Kaplan-Meier survival curves of 31 patients with primary intrathoracic liposarcoma. OS, overall survival; PFS, progression-free survival; LPFS, local progression-free survival

The median follow-up time in this study was 77 months (range 10–403 months). Disease progression appeared in 26 (83.9%) patients, including 20 (64.5%) with local recurrence and 11 (35.5%) with distant metastasis. Thirteen patients developed local recurrence only once with a median interval of 28 months (range 2–160 months), 4 developed twice with a median interval of 30 months (range 8–120 months), and 3 developed three times with a median interval of 15 months (range 12–24 months). Eleven (55.0%) patients with local recurrence underwent surgery. The median local progression-free survival (LPFS) was 44 months (range 2–214 months). The 3- and 5-year LPFS rates were 53.8% and 37.2% (Fig. 1). All the patients diagnosed with myxoid, dedifferentiated, and pleomorphic liposarcomas showed either local recurrence or distant metastasis. All the patients diagnosed with dedifferentiated liposarcomas showed local progression. Burt et al. [3] recommended continuous exploration of aggressive adjuvant treatment in consideration of the high local recurrence rate (64.0%) even after radical resection. Hahn et al. [5] reported that approximately 40% of mediastinal liposarcomas recurred after surgery and multiple successive recurrences were common.

In the present study, 18 (58.1%) patients died of disease progression: 10 died of local recurrence and 8 died of distance metastasis. Our results showed that adjuvant radiotherapy for patients with primary intrathoracic liposarcoma remained controversial. Chan et al. [9] reported that it was essential to completely resect the primary intrathoracic liposarcomas as the radical cure of this disease.

The median overall survival (OS) in the present study was 102 months, and the 3- and 5-year OS rates were 76.2% and 68.9%. The median progression-free survival (PFS) was 30 months, and the 3- and 5-year PFS rates were 40.7% and 23.8% (Fig. 1). The median survival of patients with myxoid, dedifferentiated, mixed, well-differentiated, and pleomorphic subtypes of primary intrathoracic liposarcoma were 120, 34, 68, 174, and 36 months, respectively.

The univariate analyses of the present study revealed associations between that histological subtypes (dedifferentiatied subtype [*P* = 0.033]) and OS (Additional file 2: Table S2) as well as between initial symptom (chest pain [P = 0.014]) and PFS (Additional file 3: Table S3). On multivariate analysis, histological subtypes (P < 0.05) and initial symptom (P < 0.05) were identified as independent prognostic factors for OS (Additional file 2: Table S2) and PFS (Additional file 3: Table S3). Chen et al. [9] reported that the histological subtype and radical surgery were the influencing factors for OS of patients with primary intrathoracic liposarcoma. The well-differentiated liposarcoma patients who underwent radical surgery had the best prognosis than the patients with other histological subtypes of liposarcoma. Klimstra et al. [2] reported that OS was associated with histological subtype and the completion of surgical resection. They found that patients with myxoid or pleomorphic liposarcoma had a poorer prognosis when compared with those with well-differentiated tumors. Burt et al. [3] reported that the long-term prognosis for patients with mediastinal liposarcomas was poor because of the late diagnosis, involvement of vital structure, and inability to achieve complete resection. Chen et al. [9] reported that shorter disease-free survival and OS were observed in patients with myxoid, dedifferentiated, and pleomorphic subtypes of primary intrathoracic liposarcoma as compared with patients with well-differentiated subtype.

In summary, histological subtype is the most important prognostic factor for OS of patients with primary intrathoracic liposarcoma. Radical surgery is the most effective treatment for primary intrathoracic liposarcoma.

## Additional files

**Additional file 1: Table S1.** Baseline demographic and clinical characteristics of 31 patients with primary intrathoracic liposarcomas.

**Additional file 2: Table S2.** Univariate and multivariate analysis of prognostic factors associated with overall survival of patients with primary intrathoracic liposarcomas.

**Additional file 3: Table S3.** . Univariate and multivariate analysis of prognostic factors associated with progression-free survival of patients with primary intrathoracic liposarcomas.

## Abbreviations

R0: complete resection
R1: microscopic residual tumor
R2: macroscopic residual tumor
LPFS: local progression-free survival
OS: overall survival
PFS: progression free survival

## References

[1] Shmookler BM, Enzinger FM (1983). Liposarcoma occuring in children. An analysis of 17 cases and review of the literature. Cancer..

[2] Klimstra DS, Moran CA, Perino G, Koss MN, Rosai J (1995). Liposarcoma of the anterior mediastinum and thymus. A clinicopathologic study of 28 cases. Am J Surg Pathol.

[3] Burt M, Ihde JK, Hajdu SI, Smith JW, Bains MS, Downey R (1998). Primary sarcomas of the mediastinum: results of therapy. J Thor Cardiovasc Surg.

[4] Moreau LC, Turcotte R, Ferguson P, Wunder J, Clarkson P, Masri B (2012). Myxoid\round cell liposarcoma (MRCLS) revisited: an analysis of 418 primarily managed cases. Ann Surg Oncol.

[5] Hahn HP, Fletcher CD (2007). Primary mediastinal liposarcoma: clinicopathologic analysis of 24 cases. Am J Surg Pathol.

[6] Dei Tos AP (2000). Liposarcoma: new entities and evolving concepts. Ann Diagn Pathol.

[7] Gladish GW, Sabloff BM, Munden RF, Truong MT, Erasmus JJ, Chasen MH (2002). Primary thoracic sarcomas. Radiographics..

[8] Unal OU, Oztop I, Yasar N, Urakci Z, Ozatli T, Bozkurt O (2015). Clinicopathologic characteristics, treatment outcomes, and prognostic factors of primary thoracic soft tissue sarcoma: a multicenter study of the Anatolian Society of Medical Oncology (ASMO). Thoracic Cancer..

[9] Chen M, Yang J, Zhu L, Zhou C, Zhao H (2014). Primary intrathoracic liposarcoma: a clinicopathologic study and prognostic analysis of 23 cases. J Cardiothor Surg.

[10] Enzinger FM, Weiss SW (1995). Soft tissue tumor.

