# Supplementary material for: Primary intrathoracic liposarcoma: a clinical analysis of 31 cases
Source: Cancer Commun (Lond). 2019 Apr 2;39:15. doi: 10.1186/s40880-019-0358-8 (PMC6444813; doi:10.1186/s40880-019-0358-8)
Supplement: Supplementary file 1 — Additional file 1: Table S1. Baseline demographic and clinical characteristics of 31 patients with primary intrathoracic liposarcomas. [file 40880_2019_358_MOESM1_ESM.docx]

**Additional Table S1.** Baseline demographic and clinical characteristics of 31 patients with primary intrathoracic liposarcomas**.**

| **Characteristics** | | **No. of patients**  **case (%)** |
| --- | --- | --- |
| Age (years) |  |  |
|  | <50 | 18 (58.1) |
|  | ≥50 | 13 (41.9) |
| Gender |  |  |
|  | Male | 19 (61.3) |
|  | Female | 12 (38.7) |
| Smoking |  |  |
|  | Yes | 5 (16.1) |
|  | No | 26 (83.9) |
| Initial symptom |  |  |
|  | Chest pain | 7 (22.6) |
|  | Chest tightness | 13 (41.9) |
|  | Cough | 5 (16.1) |
|  | Asymptomatic | 6 (19.4) |
| Histological subtype |  |  |
|  | Myxoid | 13 (41.9) |
|  | Well-differentiated | 6 (19.4) |
|  | Mixed-type | 5 (16.1) |
|  | Pleomorphic | 4 (12.9) |
|  | Dedifferentiated | 3 (9.7) |
| Tumor location |  |  |
|  | Medianstinum | 24 (77.4) |
|  | Pleura or pericardium | 5 (16.1) |
|  | Pulmonary | 2 (6.5) |
| Tumor diameter |  |  |
|  | <10 cm | 14 (45.2) |
|  | ≥10 cm | 17 (54.8) |
| Treatment |  |  |
|  | Surgery alone | 17 (54.8) |
|  | Radiotherapy alone | 3 (9.7) |
|  | Surgery + radiotherapy | 8 (25.8) |
|  | Surgery + chemotherapy | 1 (3.2) |
|  | Surgery + radiotherapy + chemotherapy | 2 (6.5) |
| Treatment nature |  |  |
|  | Radical therapy | 21 (67.7) |
|  | Palliative therapy | 10 (32.3) |
