# Supplementary material for: Primary intrathoracic liposarcoma: a clinical analysis of 31 cases
Source: Cancer Commun (Lond). 2019 Apr 2;39:15. doi: 10.1186/s40880-019-0358-8 (PMC6444813; doi:10.1186/s40880-019-0358-8)
Supplement: Supplementary file 2 — Additional file 2: Table S2. Univariate and multivariate analysis of prognostic factors associated with overall survival of patients with primary intrathoracic liposarcomas. [file 40880_2019_358_MOESM2_ESM.docx]

**Additional Table S2.** Univariate and multivariate analysis of prognostic factors associated with overall survival of patients with primary intrathoracic liposarcomas.

| **Parameter** | **Univariate analysis** | ***P* value** | **Multivariate analysis** | ***P* value** |
| --- | --- | --- | --- | --- |
|  | **HR (95% CI)** |  | **HR (95% CI)** |  |
| Gender (male *vs.* female) | 0.678 (0.305-1.504) | 0.339 |  |  |
| Age (<50 *vs.* ≥50) | 1.109 (0.504-2.441) | 0.798 |  |  |
| Smoking | 0.900 (0.307-2.639) | 0.848 |  |  |
| Initial symptom (chest pain *vs.* chest tightness/cough/aymptomatic) | 0.272 (0.096-0.771) | 0.014 | 0.103 (0.021-0.501) | 0.005 |
| Histological subtype (well-differentiated *vs.* myxoid/mixed-type/pleomorphic/dedifferentiated) | 0.251 (0.071-0.893) | 0.033 | 0.137 (0.037-0.511) | 0.003 |
| Tumor location (pulmonary *vs.* mediastinum/pleura) | 3.126 (1.066-9.160) | 0.038 | 0.553 (0.131-2.339) | 0.420 |
| Tumor diameter (<10 *vs.* ≥10cm) | 1.061 (0.487-2.310) | 0.881 |  |  |
| Surgery | 1.388 (0.412-4.678) | 0.597 |  |  |
| Radiotherapy | 1.258 (0.580-2.731) | 0.561 |  |  |
| Combined therapy | 1.060 (0.467-2.410) | 0.889 |  |  |
| Treatment nature (radical therapy *vs.* palliative therapy) | 1.080 (0.479-2.434) | 0.853 |  |  |

HR, hazard ratio; CI, confidence interval.
